# Pore structure characteristics and methane adsorption and desorption properties of marine shale in Sichuan Province, China

**DOI:** 10.1039/c7ra11846e

**Published:** 2018-02-08

**Authors:** Yue Changtao, Li Shuyuan, Wen Hailong, Yang Fei, Xu xinyi

**Affiliations:** College of Science, China University of Petroleum Beijing 102249 China yuect@cup.edu.cn +86-10-89735669

## Abstract

Shale gas is one of the most promising resources for unconventional natural gas. Several shale samples were collected from the Silurian Longmaxi Formation in the Yibin region, Sichuan Province, China. The basic geological parameters of the shale samples including total organic carbon, clay mineral content, and vitrinite reflectance were detected. Pore structure characteristics were analyzed with scanning electron microscopy, high-pressure mercury injection, and low-temperature nitrogen and carbon dioxide adsorption methods. Isothermal adsorption and desorption experiments were carried out using gravimetric methods. The isosteric heat of the shale adsorption was calculated using the isothermal adsorption experimental results. According to the experimental results, the shale samples have high maturity, low porosity and penetration. The surface morphological structures include organic pores, clay mineral pores, intergranular pores of authigenic minerals, dissolution pores and micro-cracks. Micropores comprised the majority of the developed pores in the shale samples and play a major role in adsorption processes. The adsorption and desorption results show that the adsorption amount of gas mainly undergoes a rapid increase phase, a slowly rising transition phase and a gentle phase, and desorption hysteresis generally occurs during gas desorption. Adsorption thermodynamics results show that the volume of adsorbed gas decreases with the increase of adsorption temperature and the isosteric heat increases with the increase of the volume of the adsorbed gas.

## Abstract 2

Isothermal adsorption and desorption experiments were carried out using gravimetric method with magnetic suspension balance.

## Graphical abstract

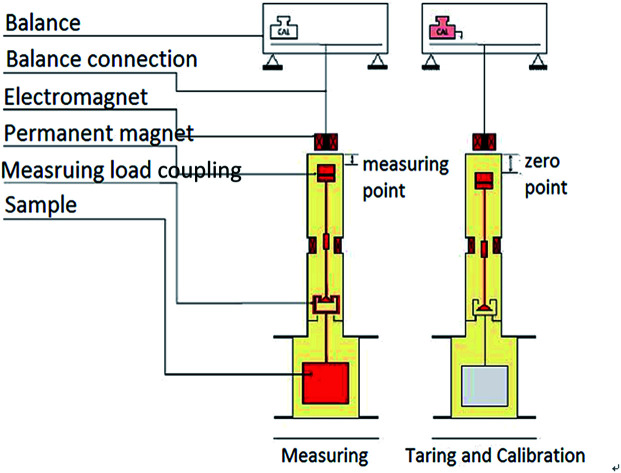

## 1 Introduction

In recent years, shale gas has received considerable attention as one of the most promising unconventional resources for natural gas. Shale gas resources are widely distributed worldwide, with the total reserves at approximately 456.23 × 10^12^ m^3^. The recoverable shale gas in China is estimated to be approximately 31 × 10^12^ m^3^.^1,2^ The Sichuan basin is regarded as the region with the most potential for exploration and exploitation in China. The Lower Silurian Longmaxi Formation reservoirs with organic-rich shale in the Sichuan basin are some of the key areas of shale gas exploration.^3–5^

Shale gas is stored in and generated from a fine-grain sedimentary rock rich in organic kerogen materials and inorganic clay minerals.^6^ Given the complex composition and multiscale structure of shale reservoirs, the total gas in shale includes free gas located within the pore spaces, adsorbed gas on the surface of organic matter or clay mineral, and a small amount of dissolved gas in organic materials.^7^ In terms of gas in place (GIP), unlike conventional natural gas reservoirs, the adsorbed gas amount plays an important role in shale reservoirs, and the adsorbed gas amount in shale reservoirs varies from 15% to 85% of the GIP.^8,9^ Moreover, the total amount of adsorbed gas in shale can be assessed with the characteristics of isothermal adsorption and desorption. Therefore, understanding the mechanism of adsorption and desorption on the surface of the shale is crucial for evaluating the reservoir and enhancing shale gas recovery.^10,11^

Previous studies have illustrated the pore structure and adsorption capacity of shale in different regions.^12–15^ Various techniques, such as X-ray diffraction (XRD), mercury intrusion, scanning electron microscopy (SEM), and N_2_ adsorption test have been utilized to detect the pore structures in shale gas samples. Mercury intrusion is an effective method to characterize the mesopore (2 nm ≤ diameter ≤ 50 nm) and macropore (diameter > 50 nm). N_2_ adsorption and carbon dioxide adsorption analysis can detect micropore (diameter < 2 nm) structure of the shale. Because of their large surface area, these micropores that exist in shale can serve the adsorption sites for methane.

In this paper, the selected shale samples are from the Silurian Longmaxi Formation in Yibin region of Sichuan Province. The basic geological parameters of the shale samples were detected, including total organic carbon (TOC), clay mineral content, and vitrinite reflectance. Pore structure characteristics were analyzed qualitatively and quantitatively through SEM, high-pressure mercury injection, low-temperature nitrogen and carbon dioxide adsorption methods. Isothermal adsorption and desorption experiments were carried out using gravimetric method. The isosteric heat of the shale adsorption were calculated based on the isothermal adsorption experimental results.

## 2 Experimental

### 2.1 Shale samples

All the shale samples, which were collected from the Silurian Longmaxi Formation in Yibin region, Sichuan Province of China, were black mud shales. Prior to measurement, 100 g of each sample was ground into powder of 60–80 mesh size. These powder samples were prepared for analysis of total organic carbon, vitrinite reflectance test, X-ray diffraction, scanning electron microscopy, high pressure mercury test and N_2_/CO_2_ adsorption.

### 2.2 TOC, vitrinite reflectance and X-ray diffraction

WR-112 LECO carbon analyzer of LECO Company was used to conduct the organic carbon analysis. The size of the sample particle is less than 0.2 mm. Iron and tungsten were added into the sample particle to aid combustion.

UMSP-50 micro spectrophotometer was used to test vitrinite reflectance. The test conditions were temperature under 26 °C, a wavelength of 546 nm ± 5 nm (green), and ×25 to ×100 unstrained oil immersion objective. One hundred tungsten halogen lamps and electronic exchange regulator of 3 kVA were also used.

The mineral composition test was done using XRD under Cu-Kα radiation. Emission and scattering slits are both 1°, and the receiving slit is 0.3 mm. The operating voltage is 30–45 kV, and the electric current is 20–100 mA; the scanning speed is 2° min^−1^, and the sampling step width is 0.02°.

### 2.3 Scanning electron microscopy

Field emission scanning electron microscope (FE-SEM) images of shale samples were collected using the Quanta 200F (FEI Instrument Corporation) equipped with an energy-dispersive spectrometer. To obtain a clear image, the shale sample slab was prepared through an Ar-ion milling instrument before FESEM imaging, producing a flat surface with minor topographical variation suitable for high-magnification imaging. SEM imaging was conducted at 35% humidity and 24 °C. To better understand the pore structure of shale samples, SEM images were assessed under different magnifications.^16^

### 2.4 Mercury intrusion

Mercury intrusion measurements were performed using the Poremaster PM-33-13 analyzer (Quantachrome Instruments Corporation). Applied pressures varied from 0.01 MPa to 80 MPa, covering the pore diameter range from approximately 10 nm to 1000 nm. The experiment was in accordance with the oil and gas industry standard of SY/T5336-2006 and SY/T5346-2005 of China. Before the experiment, samples of shale blocks were prepared and cut into cylindrical shapes. The cylindrical shale samples were oven dried at 105 °C to a constant weight to remove volatile matters. Pore structure parameters were calculated by measuring the volume of mercury that had been injected into the shale samples. Washburn formula was used to calculate the pore radius of the shale samples.^15,17^

### 2.5 Liquid nitrogen adsorption

Liquid nitrogen adsorption is commonly used for characterizing the mesopores of shale samples. The Quadrasorb evo of Quantachrome was used to test mesopores structure and part of masopores structure by N_2_ adsorption method. Before the test, all the samples were constant temperature dried under 105 °C and vacuum, the adsorption gas purity is 99.99% and the experimental temperature is 77.35 K. Specific surface area and volume of mesopores were determined using nonlocal density functional theory (NLDFT) method.

### 2.6 Carbon dioxide adsorption

Analysis of carbon dioxide adsorption is an effective method for detecting micropores. Carbon dioxide adsorption was measured using the Autosorb-iQ analyzer (Quantachrome Instruments Corporation). Before the measurement, moisture and volatile compounds were removed from the sample powder (60–80 mesh) through a vacuum environment at 105 °C for 24 h. The surface area and volume of the micropores were also determined with NLDFT method.

### 2.7 Isothermal adsorption and desorption experiment

**Fig. 1:**
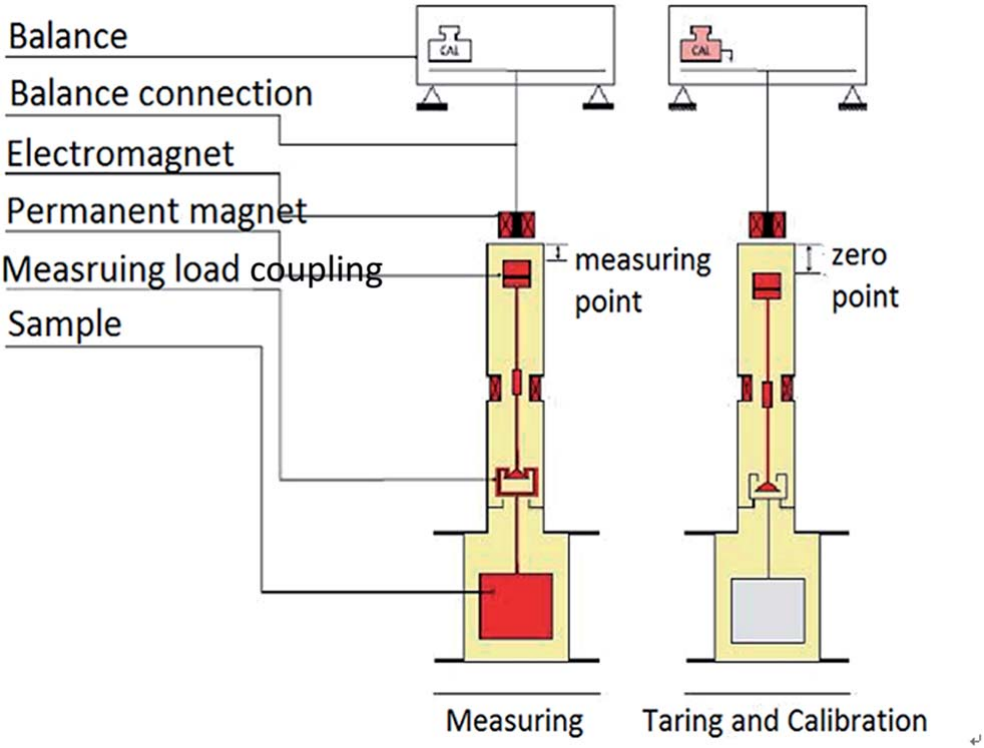
Working principle of magnetic suspension balance used in isothermal adsorption.

The adsorption capacity of methane was measured with the gravimetric method using magnetic suspension balance (MSB, Fig. 1). The weight of the shale sample was balanced using a non-contact suspending coupling mechanism. The balance has zero point and measuring point, and the two states automatically switch regularly to remove the inherent negative effects from zero drift effectively (reaching 0.00001 g accuracy). This design can achieve high precision measurement. This experiment was divided into four procedures: sample pretreatment, buoyancy experiment; adsorption and desorption experiment.

The aim of pretreatment is to eliminate the effect of water and other gases during the adsorption process, and then the condition is that shale samples were heated at 105 °C and were vacuumed to balance weight. The heating temperature is suitable for removing free water and adsorbed water, not destroying the combined water. The impurity gases in the shale samples can be removed in a vacuum state. After the pretreatment, the buoyancy test was performed with helium to calibrate the buoyancy of samples and container suffered, which can calculate the adsorption volume accurately.

The adsorption experiment temperature was 30 °C, and shale samples were pulverized to 0.18 mm. Pressure points were selected as 0 MPa, 0.5 MPa, 1 MPa, 2 MPa, 4 MPa, 6 MPa, 10 MPa, 15 MPa, 20 MPa, 25 MPa, and 30 MPa, respectively. Shale sample pretreatment was performed in a vacuum environment at 105 °C. The obtained adsorbed gas was methane at 99.99% purity.

The desorption experiment was similar to the adsorption experiment, except that the pressure points were setup in the opposite order, that is, 30 MPa, 25 MPa, 20 MPa, 15 MPa, 10 MPa, 6 MPa, 4 MPa, 2 MPa, 1 MPa, 0.5 MPa, and 0 MPa, respectively.

## 3 Result of geologic parameter and pore structure

### 3.1 Geologic parameter

The results of geologic parameter of shale samples are shown in Table 1. As shown in Table 1, TOC of shale samples ranged between 1.92% and 3.38% with the increasing average depth. Vitrinite reflectance of shale samples is approximately 2%, which indicates that the shale samples have high maturity. XRD result showed that shale samples have high clay mineral content that is favourable for the adsorption capacity of shale.

**Table 1:** Results of geologic parameter analysis of shale samples

Number	Sedimentary environment	Average depth (m)	TOC (%)	Vitrinite reflectance (%)	Clay mineral content (%)
1	Marine	2429.09	1.92	1.94	54.5
2	Marine	2436.34	2.04	2.15	45.1
3	Marine	2440.90	3.38	1.96	30.9

### 3.2 Scanning electron microscopy

**Fig. 2:**
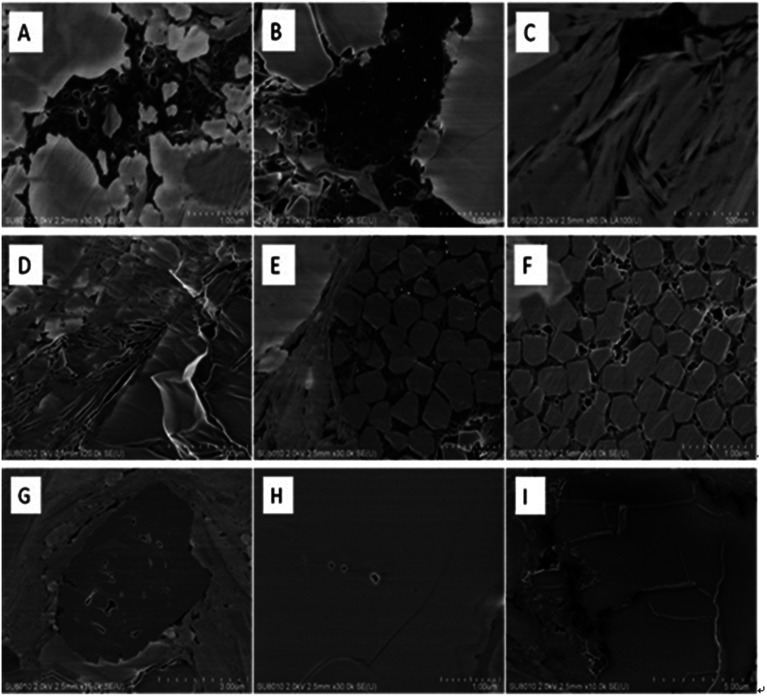
SEM micrograph of pores in shale samples.

The results of SEM are listed in Fig. 2. The surface morphological structures include organic pores, clay mineral pores, intergranular pores of authigenic minerals, dissolution pores and micro-cracks. Organic pores (Fig. 2A and B), most of which are amorphous, predominantly developed in the shale sample. A large amount of pores (Fig. 2C and D) developed on the clay mineral because of the pressure from diagenetic process. Some pores also developed between authigenic mineral particles such as pyrite (Fig. 2E and F). Dissolved pores are always triangular and square in shape (Fig. 2G and H) and are closely related to the dissolution of acidic fluids. Shale gas mainly seeps through micro fractures, which are randomly distributed in the surface of shale samples (Fig. 2I). The micro fractures can be several micrometers long and several nanometers wide.^18–20^

### 3.3 Mercury injection

Table 2 shows the macropore structure parameters of shale samples. Porosity and penetration of shale samples are relatively low, which is in accordance with the characteristic of low porosity and low permeability of shale as the dense rock.^21^ The average pore radius of macropore was approximately 0.17–0.20 mm, in the range of low-permeability reservoir. The low permeability indicates that the shale gas is not easy to flow inside the shale, which is beneficial to the storage of shale gas. Saturation of advanced mercury was approximately 20%, and the efficiency of mercury ejection was relatively high at 70%.

**Table 2:** Results of mercury test of shale samples

Number	Penetration (10^−3^ μm^2^)	Porosity (%)	Mercury saturation (%)	Ejection efficiency (%)	Pore radius (μm)
1	0.002	3.15	21.41	78.60	0.20
2	0.005	3.22	22.65	68.00	0.18
3	0.001	3.18	17.02	74.17	0.17

### 3.4 Liquid nitrogen adsorption

**Fig. 3:**
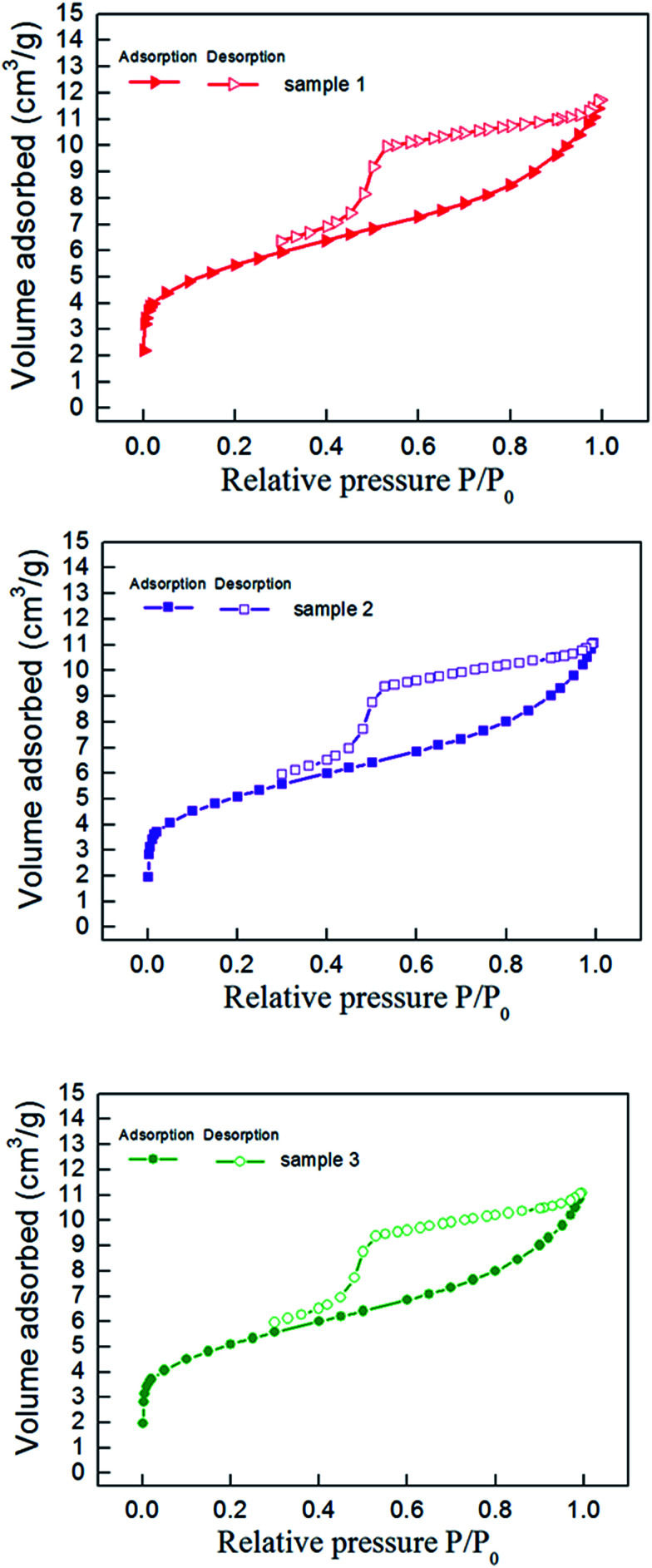
Nitrogen adsorption–desorption isotherms of shale samples.

**Fig. 4:**
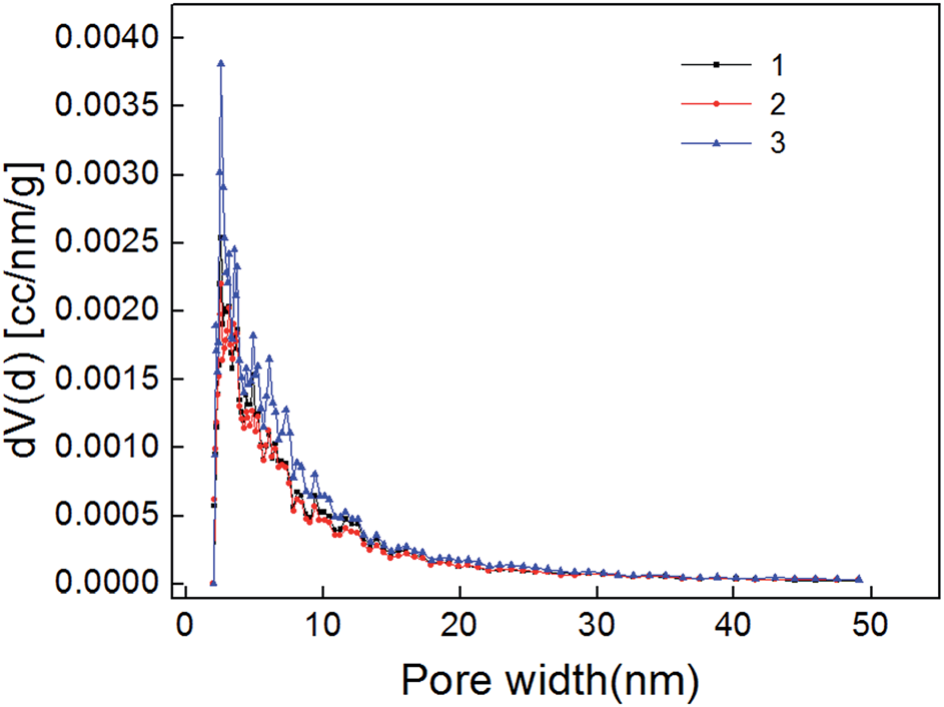
Mesopore distribution of shale samples.

The nitrogen adsorption–desorption curve of shale samples is shown in Fig. 3. The curve belongs to type IV adsorption isotherm, indicating that mesopore developed in shale samples.^22^ Mesopore structure parameter determined with NLDFT method is listed in Table 3. Pore size distribution of shale samples is shown in Fig. 4. According to the results, the pore volume ranged from 0.016–0.021 cm^3^ g^−1^ and the specific surface area of mesopores ranged from 19.84–30.53 m^2^ g^−1^. In the 0–50 nm range, we can find that the pore size mainly distribute in 0–10 nm, especially in 0–2 nm. And the pore volume of shale samples decreased gradually with increased pore diameter in the shale.

**Table 3:** Mesopore parameters of shale samples

Number	Surface area (m^2^ g^−1^)	Pore volume (cm^3^ g^−1^)	Average radius (nm)
1	22.33	0.017	1.33
2	19.84	0.016	1.43
3	30.53	0.021	1.33

### 3.5 Carbon dioxide adsorption

**Fig. 5:**
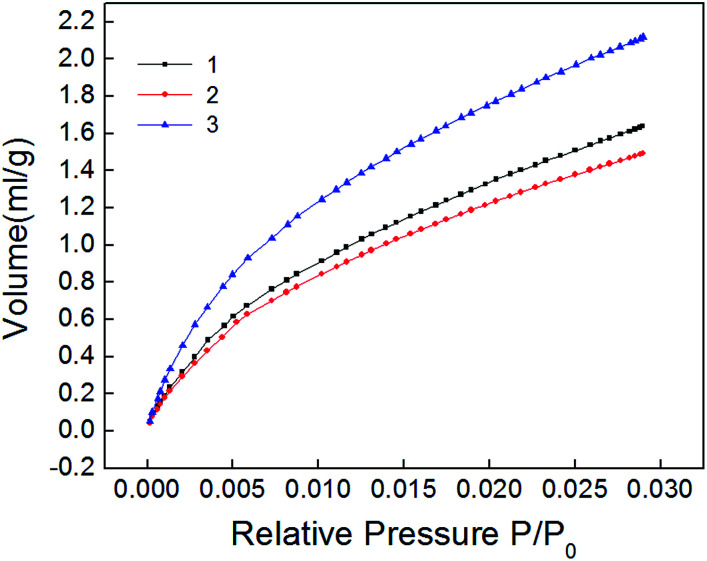
Isotherms of Carbon dioxide adsorption of shale samples.

**Fig. 6:**
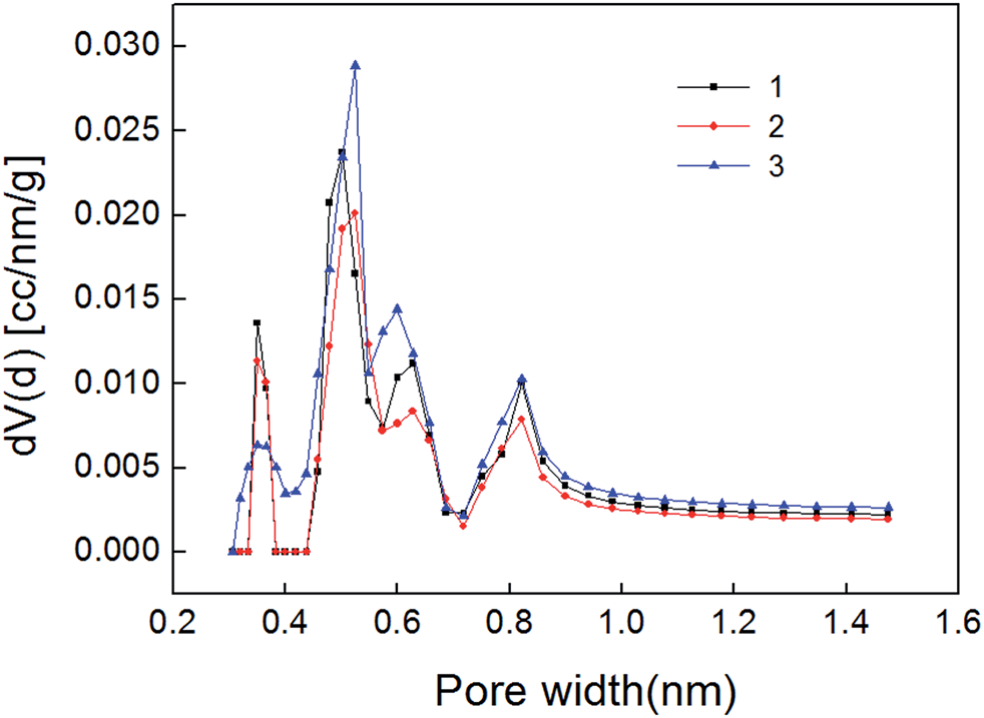
Micropore distribution of shale samples.

The carbon dioxide adsorption curve of shale samples is shown in Fig. 5. As shown, the curve belongs to type I adsorption isotherm, indicating that micropore is the main pore type of shale samples. Micropore structure parameter calculated also with NLDFT method is shown in Table 4. Pore size distribution of shale samples is shown in Fig. 6. The pore volume ranged from 0.005–0.007 cm^3^ g^−1^ and the specific surface area of micropores ranged from 15.668–22.291 m^2^ g^−1^. In the pore diameter distribution range of 0–2 nm in Fig. 6, it can be seen that the micropore diameter of shale samples is mainly distributed near 0.5 nm, and also has a certain distribution near 0.35 nm and 0.85 nm. Micropore comprised the majority of developed pores in shale samples and plays a major role in adsorption process.^23,24^

**Table 4:** Micropore parameters of shale samples

Number	Surface area (m^2^ g^−1^)	Pore volume (cm^3^ g^−1^)	Average radius (nm)
1	15.668	0.005	0.524
2	17.283	0.006	0.501
3	22.291	0.007	0.524

## 4 Isothermal adsorption and desorption experiment

### 4.1 Isothermal adsorption experiment

**Fig. 7:**
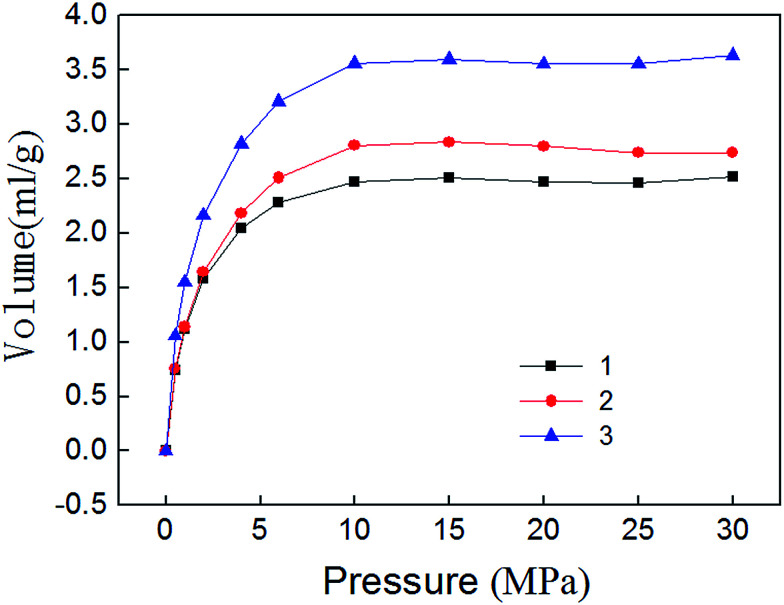
Isothermal adsorption curve of shale samples.

Isothermal adsorption experiments results are shown in Fig. 7. It can be seen that the adsorption of methane mainly experienced three stages. Adsorption volume increased rapidly during 0–10 MPa, indicating that micropores play a major role. Methane molecules can adsorb large energy, the adsorption volume sharply enlarges. This phenomenon is called micropore filling. Adsorption volume increased slowly during 10–15 MPa. Because the methane molecules strong adsorption in micropores is basically saturated, the mesopores begin to assume the major role, and the adsorption capacity become weak. Adsorption volume reached saturation after 15 MPa. The existence state of methane molecules in macropores is mainly free state. Therefore, the amount of adsorbed gas will no longer change.

### 4.2 Isothermal desorption experiment

**Fig. 8:**
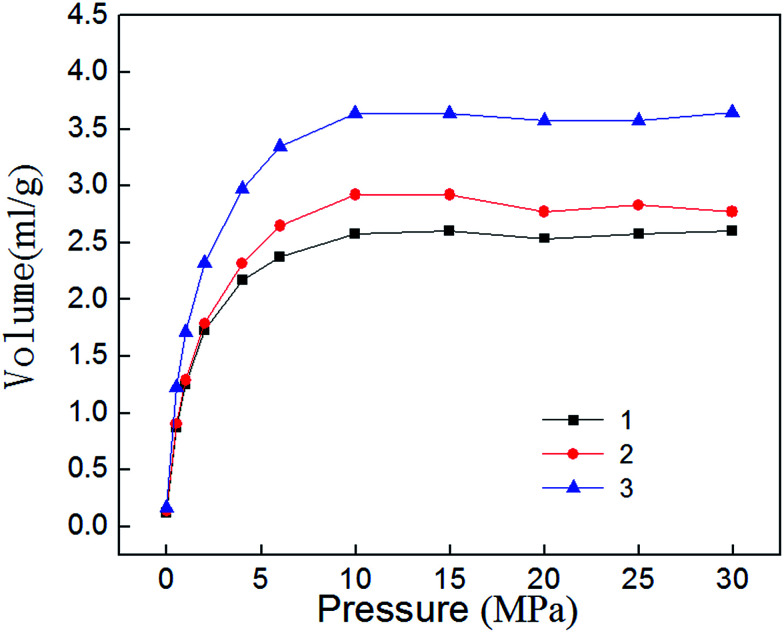
Isothermal desorption curve of shale samples.

**Fig. 9:**
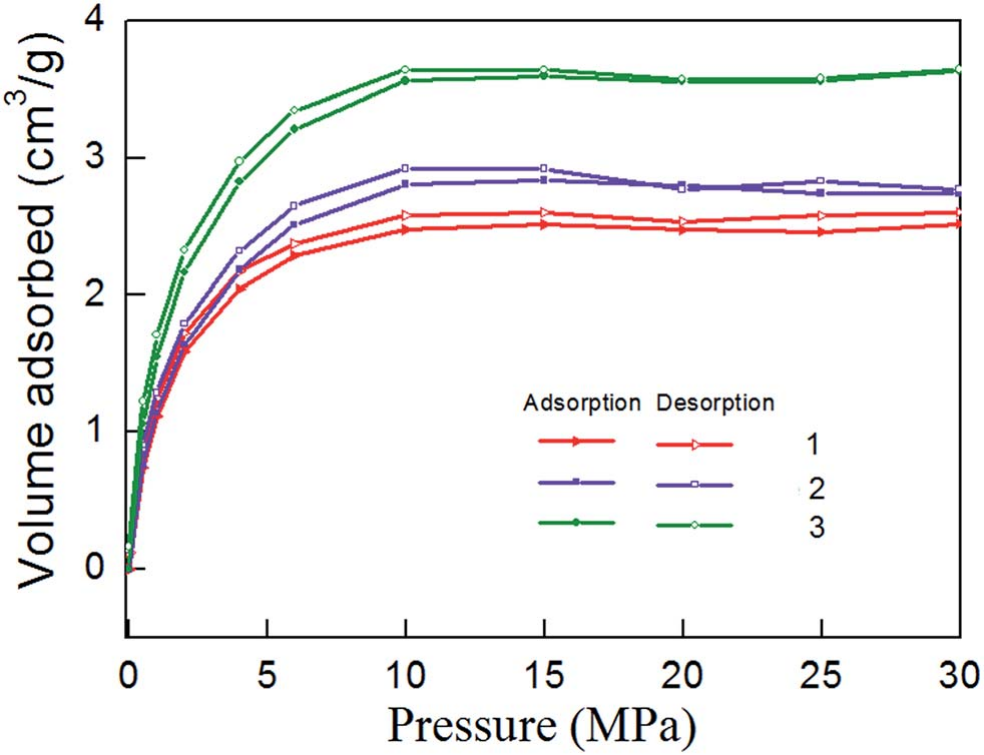
Comparison of isothermal adsorption–desorption curves.

Isothermal desorption experiment is the inverse process of adsorption, and it begins immediately after completion of the adsorption experiment. Fig. 8 shows the isothermal desorption curve, which belongs to type I adsorption isotherm in keeping with the adsorption curve. When the pressure is high (30–10 MPa), desorption velocity is small and remains at a relatively stable level. Desorption rate rises rapidly when pressure falls to 10 MPa, indicating that 10–0.5 MPa is the main period of shale gas desorption. By comparing the desorption curves with the adsorption curves, Fig. 9 can be obtained. The Fig. 9 show that shale adsorption and desorption curves did not coincide, and desorption curve is in hysteresis. Mesopores and micropores have significant binding capacity for gas molecules, coupled with the capillary condensation effect of shale matrix solid, thus eventually leading to their capability for methane desorption hysteresis.

## 5 Adsorption thermodynamics analysis

### 5.1 Influence of temperature on shale adsorption

**Fig. 10:**
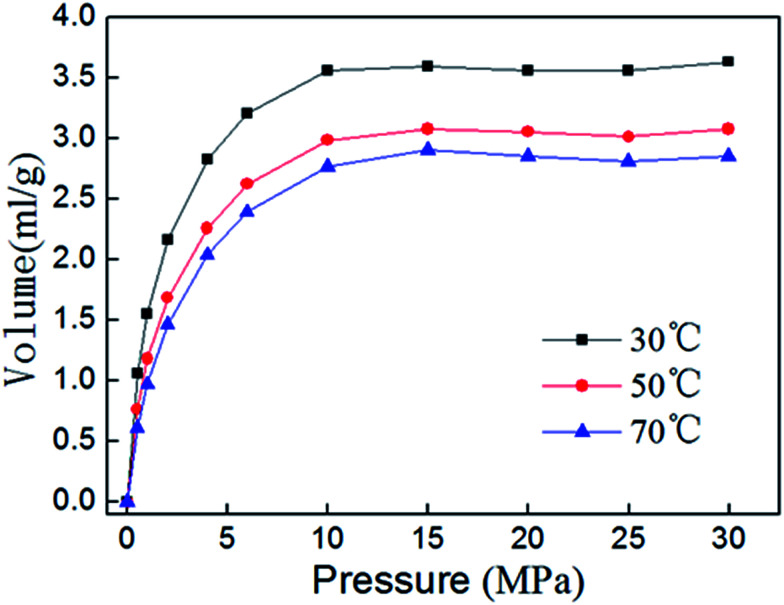
Isothermal adsorption curve of sample 3 at different temperatures.

Isothermal adsorption experiment results of shale sample 3 at different temperatures are shown in Fig. 10. The volume of adsorbed gas decreases with the increasing of adsorption temperature, thus the adsorption process can proceed more easily at lower temperature. With the increasing of adsorption temperature, potential energy should increase when methane molecules move from bulk gas phase to the surface of shale. In theory, the increase in temperature would benefit the adsorption. However, the thermal motion of methane molecules, which enable methane leave more easily from surface of shale, also rise up with the increase of temperature, and will play a greater influence on the decrease the adsorption capacity.

### 5.2 Isosteric heats of adsorption

Isosteric heat is released when a gas molecule has been adsorbed at a specific volume. Clausius–Clapeyron equation is usually used to calculate the isosteric heat^25,26^ and is expressed as follows:1
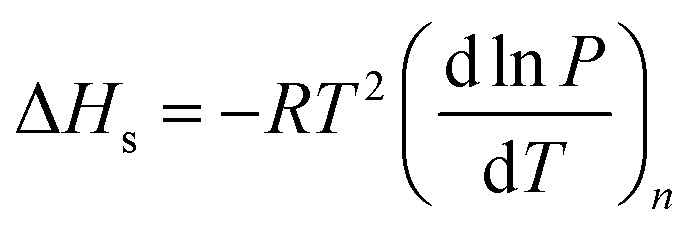
where Δ*H*_s_ is the isosteric heat (kJ mol^−1^), and *P* is the pressure (MPa). When the volume is specific, the equation can be simplified by integrating the temperature, and the new equation is2
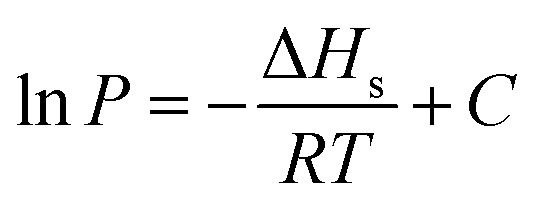


**Fig. 11:**
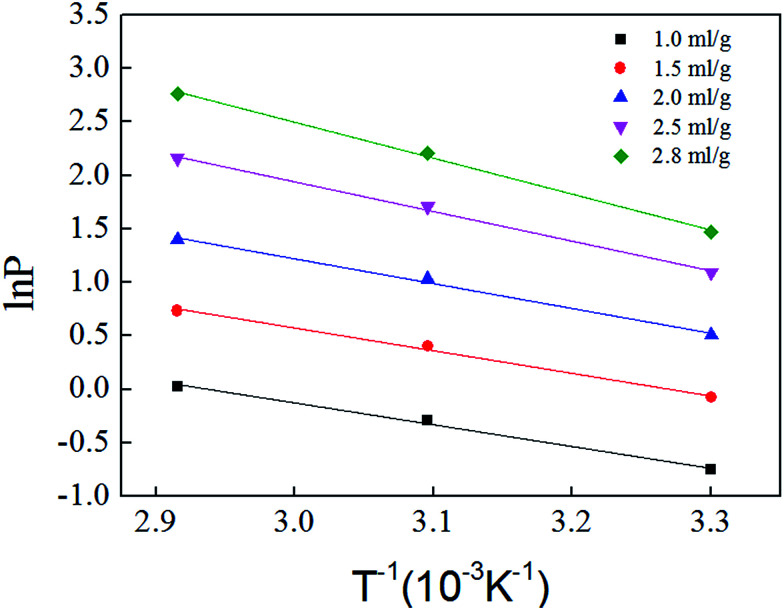
Adsorption isosteres of methane on shale samples.

Fig. 11 is based on logarithm of pressure (ln *P*) and the reciprocal of temperature (*T*^−1^). Isosteric heat of adsorption was calculated with the slope shown in Fig. 11, and the result is shown in Table 5. The average isosteric heat of adsorption was approximately 20.93 kJ mol^−1^ and is far less than that in chemistry process, which indicate that the adsorption is a physical process. Isosteric heat increases with the increasing of adsorbed gas volume.^27,28^

**Table 5:** Isosteric heat of gas adsorption on shale samples

Volume (mL g^−1^)	Slope	Intercept	Isosteric heat (kJ mol^−1^)
1.0	−2.0343	5.9585	16.91
1.5	−2.1146	6.8997	17.58
2.0	−2.3155	8.1493	19.25
2.5	−2.7685	10.2282	23.02
2.8	−3.3556	12.5468	27.90

The intermolecular forces and energy on shale surface can be used to explain the relationship between adsorbed gas volume and isosteric heat. The intermolecular forces will increase with the increasing of adsorbed gas volume. However, energy on different shale surface is not even. When adsorption happens at first in micropore, gas molecules were adsorbed at high energy position and the activation energy is low, and then the heat release is high. When the adsorption continues, gas molecules become saturated in micropores and they were adsorbed at low energy position in mesopore. The activation energy is relatively high, and the heat release is low. Isosteric heat increases with the increasing of adsorbed gas volume means that intermolecular forces has stronger effect than energy heterogeneity.

## 6 Conclusion

(1) The shale samples are all black mud shale, and they have high maturity, low porosity and penetration. The surface morphological structures include organic pores, clay mineral pores, intergranular pores of authigenic minerals, dissolution pores and micro-cracks.

(2) The average pore radius of macropore was approximately 0.17–0.20 mm. The pore volume of mesopores ranged from 0.016–0.021 cm^3^ g^−1^ and the specific surface area of mesopores ranged from 19.84–30.53 m^2^ g^−1^. The pore volume of micropore ranged from 0.005–0.007 cm^3^ g^−1^ and the specific surface area of micropores ranged from 15.668–22.291 m^2^ g^−1^. Micropore comprised the majority of developed pores in shale samples and plays a major role in adsorption process.

(3) The adsorption amount of gas mainly undergoes the rapid increase phase, the slowly rising transition phase and the gentle phase. The desorption hysteresis generally occurs during gas desorption. 10–0.5 MPa is the main period of shale gas desorption.

(4) The volume of adsorbed gas decreases with the increasing of adsorption temperature and the isosteric heat increases with the increasing of adsorbed gas volume.

## Conflicts of interest

There are no conflicts to declare.

## Supplementary Material

## References

[cit1] Hay D. (2012). Petrol. Explor. Dev..

[cit2] Zhang D. (2011). Nat. Gas. Ind..

[cit3] Dong D. Z., Zou C. N., Zhong L. J., Wang S. J., Jing L. X., Wang Y. M., Hua L. D. (2011). Geol. Bull. China.

[cit4] Jian Z. L., Dong D. Z., Chen G. S., Wang S. Q., Cheng K. M. (2009). Nat. Gas. Ind..

[cit5] Liu H. L., Wang H. Y., Liu R. H., Zhao Q., Lin Y. J. (2010). Acta Geol. Sin..

[cit6] Javadpour F., Farshi M. M., Amrein M. (2012). J. Can. Pet. Technol..

[cit7] Zhang T., Ellis G. S., Ruppel S. C., Milliken K., Yang R. (2012). Org. Geochem..

[cit8] Zhang X., Lu X. C., Zhang L., Liu Q. (2010). Adv. Earth Sci..

[cit9] Curtis J. B. (2002). AAPG Bull..

[cit10] Zhao T., Ning Z., Zeng Y. (2014). Xinjiang Pet. Geol..

[cit11] Grieser W. V. , Predicting Production Outcome From Multi-stage, Horizontal Barnett Completions, Society of Petroleum Engineers, 2009, p. 120271

[cit12] Chalmers G. R., Bustin R. M., Power I. M. (2012). AAPG Bull..

[cit13] Ambrose R. J. , Hartman R. C., Campos M. D., Akkutlu I. Y. and Sondergeld C., New Pore-scale Considerations for Shale Gas in Place Calculations, Society of Petroleum Engineers, 2010, p. 131772

[cit14] Curtis M. E. , Ambrose R. J., Sondergeld C. H. and Rai C. S., Structural characterization of gas shales on the micro-and nano-scales, Society of Petroleum Engineers, 2010, p. 137693

[cit15] Loucks R. G., Reed R. M., Ruppel S. C., Jarvie D. M. (2009). J. Sediment. Res..

[cit16] Ross D. J. K., Bustin R. M. (2007). Fuel.

[cit17] Bustin R. M. , Bustin A. M. M., Cui A., Ross D. and Pathi V. M., Impact of Shale Properties on Pore Structure and Storage Characteristics, Society of Petroleum Engineers, 2008, p. 119892

[cit18] Fishman N. S., Hackley P. C., Lowers H. A., Hill R. J., Egenhoff S. O., Eberl D. D., Blum A. E. (2012). Int. J. Coal Geol..

[cit19] Guo X., Li Y., Liu R., Wang Q. (2014). Nat. Gas. Ind..

[cit20] Guo T., Zhang H. (2014). Petrol. Explor. Dev..

[cit21] Angulo R. F. , Alvarado V. and Gonzalez H., Fractal Dimensions from Mercury Intrusion Capillary Tests, Society of Petroleum Engineers, 1992, p. 23695

[cit22] Barrett E. P., Joyner L. G., Halenda P. P. (2014). J. Manage. Eng..

[cit23] Lowell S., Shields J. E., Thomas M. A., Thommes M. (2006). Particle Technology.

[cit24] Neimark A. V., Ravikovitch P. I. (2001). Microporous Mesoporous Mater..

[cit25] Madani S. H., Sedghi S., Biggs M. J., Pendleton P. (2015). ChemPhysChem.

[cit26] Terzyk A. P., Rychlicki G. (1999). J. Therm. Anal. Calorim..

[cit27] Errais E., Duplay J., Elhabiri M., Khodja M., Ocampo R., Guyot R. B., Darragi F. (2012). Colloids Surf., A.

[cit28] Rychlicki G., Terzyk A. P. (1995). J. Therm. Anal. Calorim..

